# Machine learning links different gene patterns of viral infection to immunosuppression and immune-related biomarkers in severe burns

**DOI:** 10.3389/fimmu.2022.1054407

**Published:** 2022-11-28

**Authors:** Peng Wang, Zexin Zhang, Rongjie Lin, Jiali Lin, Jiaming Liu, Xiaoqian Zhou, Liyuan Jiang, Yu Wang, Xudong Deng, Haijing Lai, Hou’an Xiao

**Affiliations:** ^1^ Department of Burns and Plastic and Cosmetic Surgery, Xi’an Ninth Hospital, Xi’an, China; ^2^ Department of Burns and Plastic and Wound Repair Surgery, Xiang’an Hospital of Xiamen University, School of Medicine, Xiamen University, Xiamen, China; ^3^ Department of Orthopedics, 900th Hospital of Joint Logistics Support Force, Fuzhou, China; ^4^ Obstetrics and Gynecology Hospital, Institute of Reproduction and Development, Fudan University, Shanghai, China

**Keywords:** burn, immunosuppression, machine learning, prognostic model, virus infection

## Abstract

**Introduction:**

Viral infection, typically disregarded, has a significant role in burns. However, there is still a lack of biomarkers and immunotherapy targets related to viral infections in burns.

**Methods:**

Virus-related genes (VRGs) that were extracted from Gene Oncology (GO) database were included as hallmarks. Through unsupervised consensus clustering, we divided patients into two VRGs molecular patterns (VRGMPs). Weighted gene co-expression network analysis (WGCNA) was performed to study the relationship between burns and VRGs. Random forest (RF), least absolute shrinkage and selection operator (LASSO) regression, and logistic regression were used to select key genes, which were utilized to construct prognostic signatures by multivariate logistic regression. The risk score of the nomogram defined high- and low-risk groups. We compared immune cells, immune checkpoint-related genes, and prognosis between the two groups. Finally, we used network analysis and molecular docking to predict drugs targeting *CD69* and *SATB1*. Expression of *CD69* and *SATB1* was validated by qPCR and microarray with the blood sample from the burn patient.

**Results:**

We established two VRGMPs, which differed in monocytes, neutrophils, dendritic cells, and T cells. In WGCNA, genes were divided into 14 modules, and the black module was correlated with VRGMPs. A total of 65 genes were selected by WGCNA, STRING, and differential expression analysis. The results of GO enrichment analysis were enriched in Th1 and Th2 cell differentiation, B cell receptor signaling pathway, alpha-beta T cell activation, and alpha-beta T cell differentiation. Then the 2-gene signature was constructed by RF, LASSO, and LOGISTIC regression. The signature was an independent prognostic factor and performed well in ROC, calibration, and decision curves. Further, the expression of immune cells and checkpoint genes differed between high- and low-risk groups. *CD69* and *SATB1* were differentially expressed in burns.

**Discussion:**

This is the first VRG-based signature (including 2 key genes validated by qPCR) for predicting survival, and it could provide vital guidance to achieve optimized immunotherapy for immunosuppression in burns.

## Introduction

According to the Global Burden of Diseases, Injuries, and Risk Factors Study, there were approximately 8.4 million burn incidents worldwide in 2019, resulting in 110,000 deaths (1). Burn emergency techniques have advanced significantly over the past 20 years, bringing about a significant reduction in burn mortality, but the burden of infection remains high (2). Infections are triggered by the accompanying immunosuppression in burn patients. Most studies focused on infections including the bacterial ones primarily caused by *Pseudomonas aeruginosa* or *Klebsiella pneumonia*. However, burn wounds are also highly susceptible to viral infections mainly due to the impaired immune responses and functions of the immune cells within the wound micro-environment (3).

Herpes simplex virus (HSV), varicella-zoster virus (VZV), cytomegalovirus (CMV), human papillomavirus (HPV), and Epstein-Barr virus (EBV) are common pathogens in burn patients with a viral infection which are mainly latent infections (3). Post-burn immunosuppression is a common pathological process, and immunosuppression increases the risk of viral reactivation. In addition, viral infections can weaken the body’s immunity, leading to increased bacterial susceptibility (4). Viral infection is hard to detect because blisters and skin damage make the skin symptoms of viral infection unobvious. In addition, severe viral infection can lead to liver failure and severe encephalitis, easily misdiagnosed as multiple organ failure in severe burns (5–7). Although viral infection is vital to prognosis, there is still a lack of prognostic and therapeutic biomarkers related to viral infection. It is of great value to study viral markers.

Current prognostic indicators have some limitations. Total body surface area (TBSA) is the most common indicator, but it ignores age and gender and cannot accurately assess complex complications such as inhalation injury (8). The ABSI and Baux scales are used at the beginning of the burn, which cannot dynamically track the progression, and cannot evaluate the state of inflammation and the patient’s immune function (9). Some inflammatory mediators and cytokines such as IL-1, IL-6, IL-8, MCP-1, and GCS-F reflect inflammation and immune function, but are still limited (10). Immunosuppression and infections are responsible for the deaths of more than 60% of patients (11, 12). Therefore, developing new markers related to immune function and prognosis is necessary. With the advancement of artificial intelligence and medical big data technology, machine learning has become part of precision medicine to validate therapeutic and prognostic biomarkers (13–15). Based on transcriptome data, unsupervised consensus clustering has been used to reveal different patterns in diabetes and cardiovascular disease (16–18), which can be used to search for similarity and heterogeneity between transcriptome data and to divide samples into groups with different prognostic clusters (14, 19–21). Random forest and LASSO are machine learning algorithms that can screen out biomarkers related to the prognosis of many genes, and have been used to screen key genes for cardiovascular diseases and other diseases (22–24). Conjoint analysis of multiple machine learning methods can improve the accuracy of prognostic biomarkers. Therefore, combining transcriptomic data and machine learning techniques is promising for developing new prognostic markers for severe burns.

**Figure 1:**
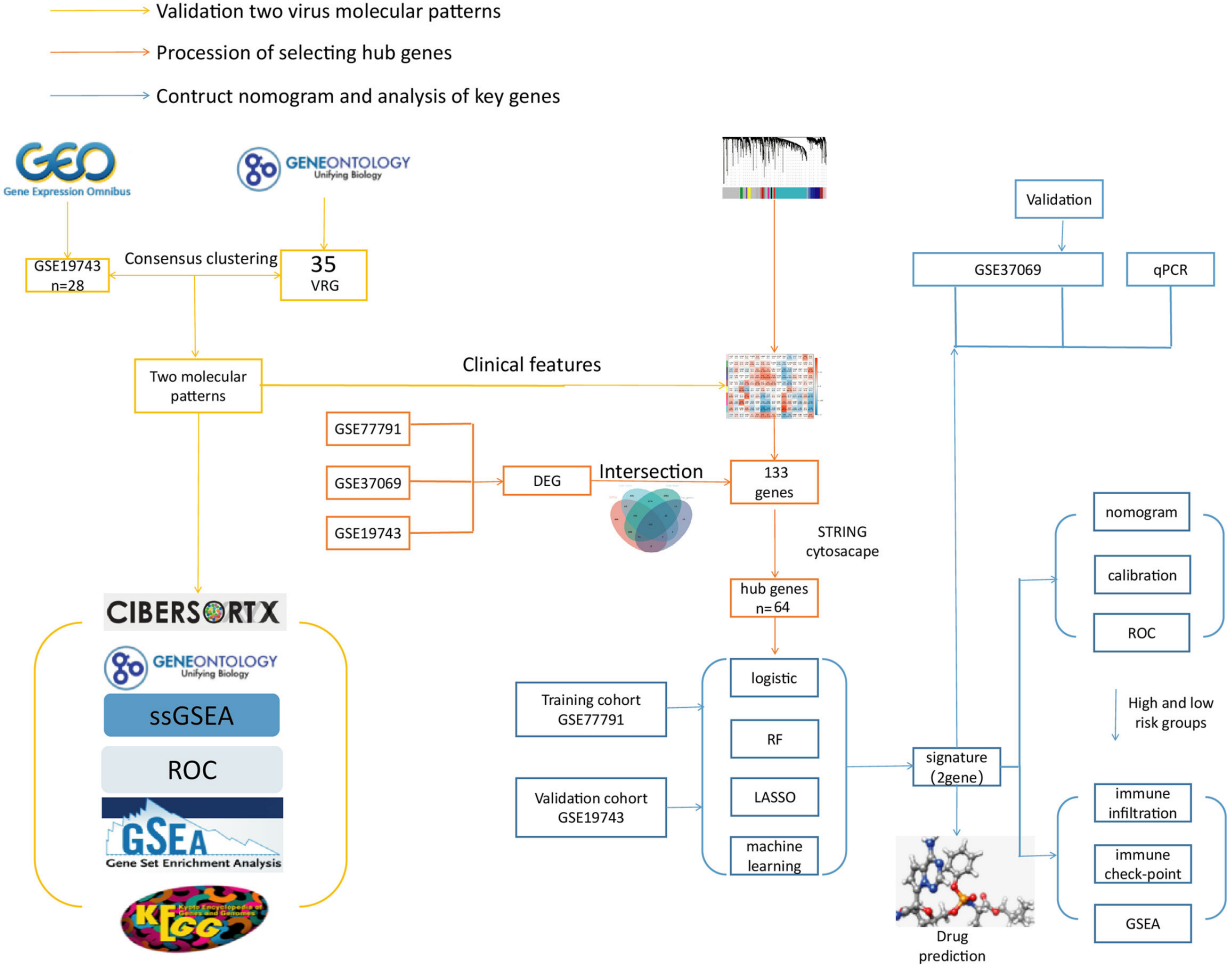
The flow chart. The yellow line is the validation of two viral molecular patterns. The orange line is the process of selecting hub genes. The blue line is the contructed nomogram and analysis of key genes.

This study used VRGs as hallmarks to identify patients grouped by two different VRGMPs by consensus clustering. Function and immune infiltration analysis between groups were assessed from four aspects: immune infiltration analysis, immune score, enrichment analysis, and clinical features. Next, in WGCNA, we identified gene sets associated with VRGMPs. The functions of these genes were fully assessed by network analysis and enrichment analysis. Further, we used RF and LASSO regression to screen for key genes associated with prognosis and constructed a nomogram by multivariate logistic regression to divide patients into high- and low-risk groups. Finally, we assessed differences in immune cells and checkpoints between patients in different risk groups and predicted potential drugs targeting key genes by molecular docking. The experimental process is shown in the flow chart (**Figure 1**).

## Methods

### Data acquisition and processing

The blood samples of burn patients were downloaded from the GEO database (GSE19743, GSE77791, and GSE37069). The patients aged 18-55, total body surface area (TBSA) >25% and sampling time after burning 7-30 days were included. Data preprocessing included transforming gene probes into gene symbols, data consolidation, and batch normalization. Probes without gene symbols or genes with more than one probe were deleted or averaged, respectively. The merged data was prepossessed by the SVA package in R software (version 4.0.5) to remove batch effects (25).

VRGs were selected from GeneOntology (GO) database (http://geneontology.org/) by keyword “Herpes simplex virus, varicella-zoster virus, cytomegalovirus, human papillomavirus, and Epstein-Barr virus”.

The *in vitro* validation cohort was obtained from GSE37069, GSE26440, and blood samples from the Department of Burns and Plastic and Cosmetic Surgery, Xi’an Ninth Hospital. Data acquisition was approved by the Ethics Committee of Xi’an Ninth Hospital (200268).

### Identification of VRGMP groups by consensus clustering

The GSE19743 dataset was included in consensus clustering analysis to explore differences in clinical traits and immunology between the different VRGMPGs. Through the k-means machine learning algorithm, the “ConsensusClusterPlus” R package was used to perform unsupervised consensus clustering, which allows for dividing or condensing cases to multiple clusters according to the provided hallmarks or signatures. Hallmarks were VRGs. In detail, we used the consensus clustering algorithm with 1,000 iterations by sampling 80% of the data in each iteration. The item-Consensus plot, the proportion of ambiguous clustering (PAC) algorithm, and the relative change in the area under the cumulative distribution function (CDF) curves confirmed the optimal cluster number. Principal component analysis (PCA) was performed to assess gene expression patterns between the VRGMPGs.

### Analysis of immune and clinical features between two VRGMPGs

The proportions of the immune cells and functions between VRGMPGs were determined by CIBERSORT, Gene Set Enrichment Analysis (GSEA), and single sample Gene Set Enrichment Analysis (ssGSEA). The ssGSEA was performed by R package “GSVA” to explore the different infiltration degrees of immune cell types, immune-related functions, and immune-related pathways between Virus 1 and 2 groups (26). GSEA software (version 3.0) was obtained from the GSEA website (http://software.Broadinstitute.org/gsea/index.Jsp), and “c2.cp.kegg.v7.4.symbols. gmt” subset was downloaded from the Molecular Signatures Database (http://www.gsea-msigdb.Org/gsea/downloads.jsp). CIBERSORT was performed online (https://cibersortx.stanford.edu/) (27). Based on gene expression profiles and VRGMPGs, the minimum gene set was set to 5 and the maximum gene set to 5000, with one thousand re-samplings, and *P* < 0.05 was considered statistically significant. The top 7 terms with the smallest p-values are shown. The prognostic value of immune cells was assessed by the receiver operating curve (ROC). We downloaded clinical information from the GSE19743 dataset to analyze clinical features (survival, ABSI, Baux, TBSA, age, sex, inhalation injury, and hospital time) between VRGMPGs.

### WGCNA and identification of VRDEGs

WGCNA is a systems biology approach that can identify modules of highly correlated genes based on linkages between gene sets and phenotypes. Gene modules associated with VRGMPGs in GSE19743 were identified using the “WGCNA” package. The “limma” package was applied to calculate the differential expression genes between healthy controls and burns in GSE19743, GSE77791, and GSE37069, respectively. We took the intersection of WGCNA module genes and burn differential genes to obtain virus-related differentially expressed genes (VRDEGs) for further analysis.

### Network analysis of VRDEGs

The functions of VRDEGs were assessed by GO and Kyoto Encyclopedia of Genes and Genomes (KEGG) enrichment analysis in The Database for Annotation, Visualization and Integrated Discovery (DAVID) (https://david.ncifcrf.gov/). We constructed a PPI network based on the STRING database (https://cn.string-db.org/), visualized it using Cytoscape, and used the MCODE plugin to identify hub genes in the network.

### Screening for prognosis-related genes

In GSE77791, univariate logistic regression analysis was performed based on the hub genes, and variables with *P* < 0.05 were used for the subsequent analysis; LASSO regression analysis was performed with the hub genes, and variables with non-zero coefficients were screened out for the next analysis; Random forest analysis was utilized to screen out the most important genes for prognosis (top 20). The results of LASSO, logistic and random forest were intersected to obtain prognostic genes for multivariate logistic regression.

### Constructing risk scoring models and independence verification

In GSE77791, multivariate logistic regression analysis was performed on prognostic genes to find key genes (*P* < 0.05). Visualize the relationship between variables and predictive models using the “rms” package. The nomogram was constructed to predict the risk of death using *CD69* and *SATB1*. Its performance was assessed by the area under the receiver operating characteristic curve (AUC), calibration curve, and decision curve. According to the nomogram risk score, patients were divided into high- and low-risk groups with a median cutoff value. To verify the independence of risk scores, univariate and multivariate logistic regression analyses were performed for risk scores, TBSA, AGE, SEX, BUAX, and ABSI, respectively.

### Immune analysis between high- and low-risk groups

Immune infiltration and immune checkpoint analysis in the high- and low-risk groups.We performed the CIBERSORT, GSEA, and ssGSEA analysis to assess immune cell expression and immune score between high- and low-risk groups. In addition, we also analyzed differences in the expression of immune checkpoint genes between high- and low-risk groups. *P* < 0.05 was considered significant. Furthermore, we performed a Pearson correlation analysis between key genes, T cell subtypes, and T cell activation/suppression.

### Drug prediction and molecular docking

Using the online network analysis tool “Networkanalysis” (https://www.Networkanalyst.ca/), the interaction network of key genes and chemicals was constructed based on the Comparative Toxicogenomics Database (CTD), and the compounds that acted on both genes at the same time were selected for the next step of molecular docking. The “.sdf” format structures of compounds were downloaded from The PubChem Project (https://pubchem.ncbi.nlm.nih.gov/). We downloaded the structures of proteins from the RSCB PDB database (https://www.rcsb.org/). PyMOL 2.2.0 software (https://pymol.org) was used to process small molecule ligands, including removal of water molecules, ligand removal, and addition of hydrogen. AutoDockTools 1.5.6 (https://autodock.scripps.edu/) was used to process receptor proteins, such as adding polar hydrogen and a charge. Molecular docking was performed by using AutoDock Vina 1.1.2 software (28). By analyzing the binding energy of the molecule, choosing the conformation with the lowest binding energy and observing the formation of hydrogen bonds, we used Pymol software to map and display the three-dimensional structures, protein residues and binding bonds of proteins.

### Validation expression of key genes

The immune system of burn patients was in dynamic changes, so we detected the expression of key genes in five different periods (0-24h, 24-72h, 72h-7d, 7d-30d, >30d) in the whole blood PCR group and the microarray group. The PCR samples (blood) were obtained from the Department of Burns and Plastic and Cosmetic Surgery, Xi’an Ninth Hospital, and data acquisition was approved by the Ethics Committee of Xi’an Ninth Hospital (202268). The Microarray group samples were collected from the public dataset (GSE37069) and do not require ethical approval. Peripheral blood mononuclear cells (PBMC) were isolated from blood using Ficoll sodium diatrizoate gradient centrifugation (Sigma-Aldrich, St. Louis, MO, USA) and were dissolved in TRIzol reagent (Invitrogen, Carlsbad, CA, USA). The total RNA was extracted using an RNeasy kit (Qiagen, Hilden, Germany) and stored at −80°C. The RR047A cDNA synthesis kit (TaKaRa, China) was used to perform the reverse-transcription of the extracted RNA, and the 2X SG Fast qPCR Master Mix (High Rox, B639273, BBI) was used for quantitative PCR of hub genes on an ABI PRISM 3700 instrument (Foster, CA, USA). GAPDH was used as an internal control, and primers are as follows:

CD69-F: 5’-ATTGTCCAGGCCAATACACATT-3’
CD69-R: 5’ –CCTCTCTACCTGCGTATCGTTTT-3’
SATB1-F: 5’-GATCATTTGAACGAGGCAACTCA-3’
SATB1-R: 5’-TGGACCCTTCGGATCACTC-3’
GAPDH-F: 5’ -TGGGTGTGAACCATGAGAAGT-3’
GAPDH-R: 5’ -TGAGTCCTTCCACGATACCAA-3’

### Statistical methods

The independent Student’s t-test was used to compare the continuous data with normal distribution, and the χ2 test for categorical data was utilized for pairwise comparisons between subgroups. The Mann–Whitney U test was used to compare differences between two independent groups when the dependent variable was either ordinal or continuous but not normally distributed. All statistical analyses were performed using the R programming language (Version 4.0.5) and SPSS software. A difference of *P* < 0.05 indicates statistical significance unless specified otherwise.

## Result

### Data acquisition and processing

**Figure 2:**
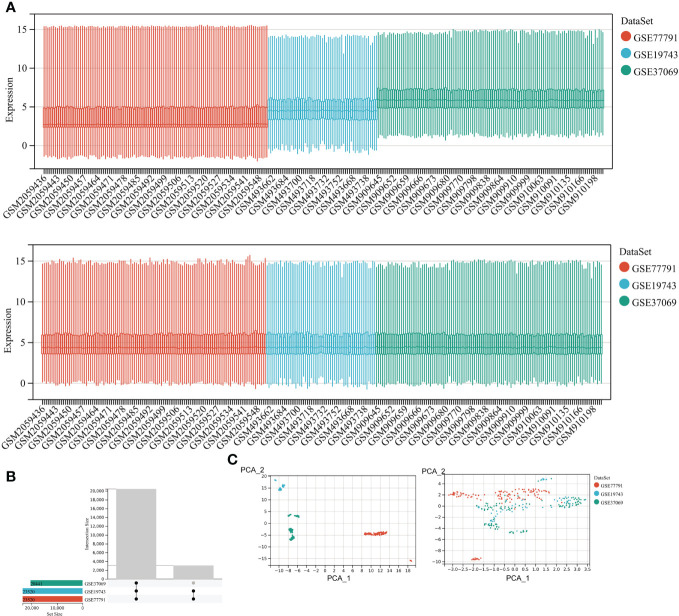
Correcting for batch effects. **(A)** Bar graphs with different colors represent different data sets. The ordinate is the expression value of the microarray data, and the abscissa is the sample number. **(B)** Overlapping genes after converting gene names between different datasets. **(C)** Sample expression profiles before batch correction and after batch correction.

We included 28 burns and 25 controls in GSE19743, 76 burns and 14 controls in GSE77791, and 83 burns and 36 controls in GSE37069. The three datasets for burns were processed with the batch effect shown below (**Figure 2**). A total of 20,441 genes were integrated from the three datasets (**Figure 2**), and the datasets were directly comparable (**Figure 2**).

Through the GO database, we extracted 35VRGs that were used to be Hallmarks in Consensus clustering.

We obtained 6 blood samples of severe burn patients (total body surface area, TBSA > 25%) aged 18-55, with sampling times including (0-24h, 24-72h, 72h-7d, 7d-30d, >30d) and 6 healthy adults with peripheral blood samples from Department of Burns and Plastic and Cosmetic Surgery, Xi’an Ninth Hospital.

Since immune cells were significantly different at different time points after burning, we selected the samples in GSE37069 to evaluate the expression of key genes at different time points, including 0-24h, 24-72h, 72h-7d, 7d-30d, >30d.

### Analysis of immune and clinical features between two VRGMPGs

**Figure 3:**
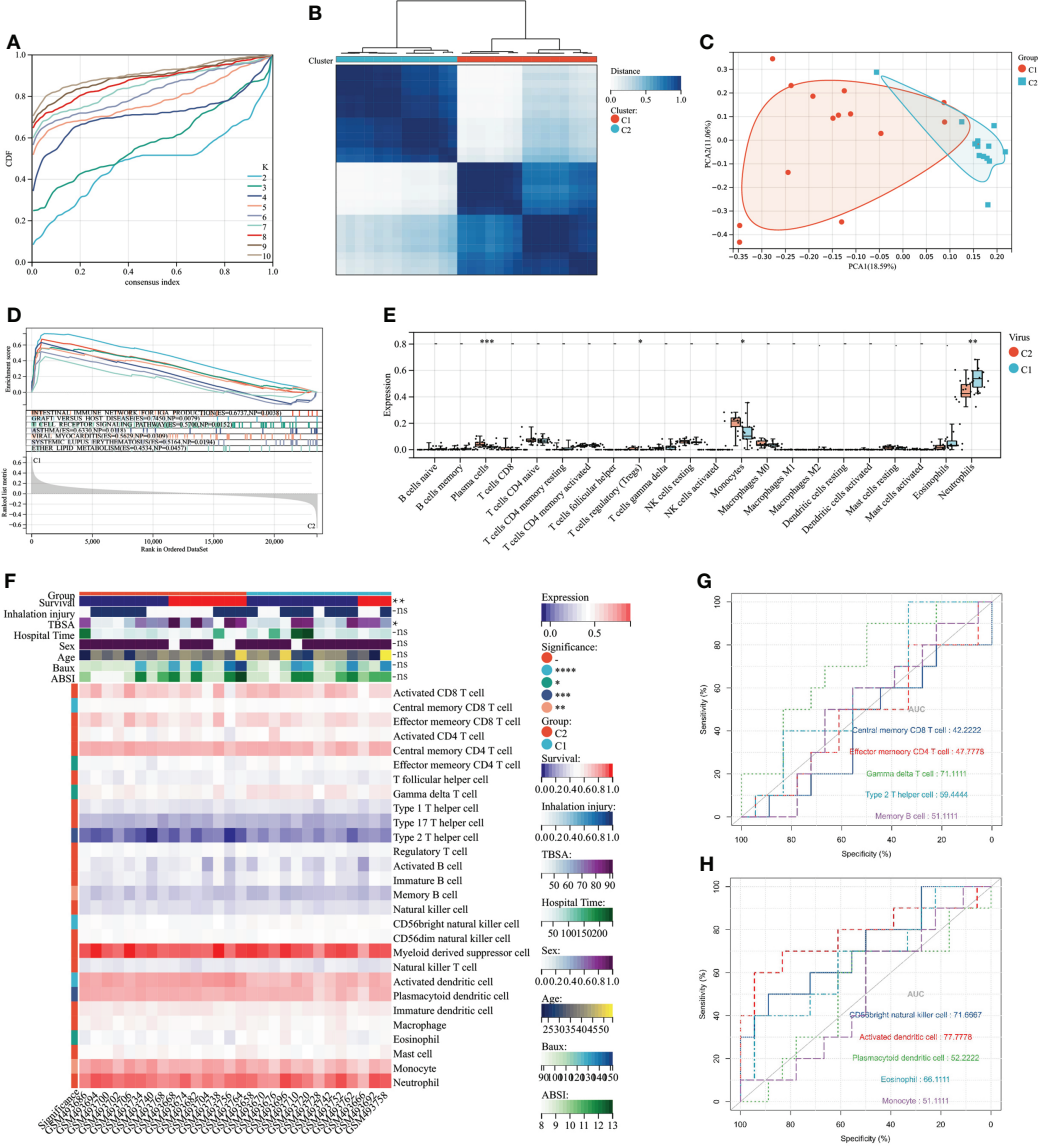
Consensus clustering and its grouping for clinical properties and immunological analysis. **(A)** Different colored lines represent different K (number of sample groups). According to the evaluation of the area under the CDF curve, the area under the CDF curve gradually increases when the K value increases. Here, the clusters with the highest average consistency in the group are the number of clusters is K=2, and the number of the next highest cluster is K=4. **(B)** When K=2, the samples can be divided into 2 groups with different expression patterns (C1 and C2). **(C)** The dots of different colors represent different groups. PCA is performed according to the gene expression data. The gene expression profiles of the two groups of patients in the figure are significantly different. **(D)** The results of GSEA showed that different colors represent enriched pathways; ES > 0 indicates that the genes of C1 are enriched in this pathway, and ES < 0 means that the genes of C2 are enriched in this pathway. **(E)** Comparison of 22 immune cells between C1 and C2 subgroups. The vertical axis represents the proportion of immune cells. Cells with *P* < 0.05 were considered to be different between groups. **(F)** ssGSEA results. In the C1 and C2 subgroups, showing differences in clinical shape, immune cells, and immune function, *P* < 0.05 was considered to be different between the different subgroups. **(G)** Immune cells and immune function ROC curves with significant differences. The larger the AUC value, the better the predictor of the patient’s prognosis. *P* < 0.05:*, *P* < 0.01:**, *P* < 0.001:***P < 0.0001:****, P > 0.05:ns.

To explore the association between severe burn patients and viral infection, we performed unsupervised clustering using genes associated with VRGs as hallmarks. Based on the machine learning algorithm “ConsensusClusterPlus”, we divided patients into two distinct virus-associated molecular patterns (C1:Virus-1 and C2:Virus-2 groups) (**Figure 3A**). According to the results of principal component analysis, different VRGMPs have different gene expression patterns (**Figure 3**). In GSEA analysis, genes in the C1 group were more enriched in INTESTINAL_IMMUNE_NETWORK_FOR_IGA_PRODUCTION, GRAFT_VERSUS_HOST_DISEASE, ASTHMA, T_CELL_RECEPTOR_ SIGNALING_PATHWAY, VIRAL_MYOCARDITIS, SYSTEMIC_LUPUS_ ERYTHEMATOSUS, and ETHER_LIPID_METABOLISM (**Figure 3**). In the CIBERSORT immune infiltration analysis, the proportions of plasma cells, Tregs, monocytes, and neutrophils significantly differed between different virus-associated molecular patterns (**Figure 3**). In ssGSEA analysis, CD8 T cells and effector CD4 T cells differed significantly between different virus-associated molecular patterns. Gamma delta T cell, CD56 bright natural killer cell, and Activated dendritic cell have better prognostic values (**Figure 3G**). TBSA and survival also differed significantly between different VRGMPGs (**Figure 3**).

### WGCNA and identification of VRDEGs

**Figure 4:**
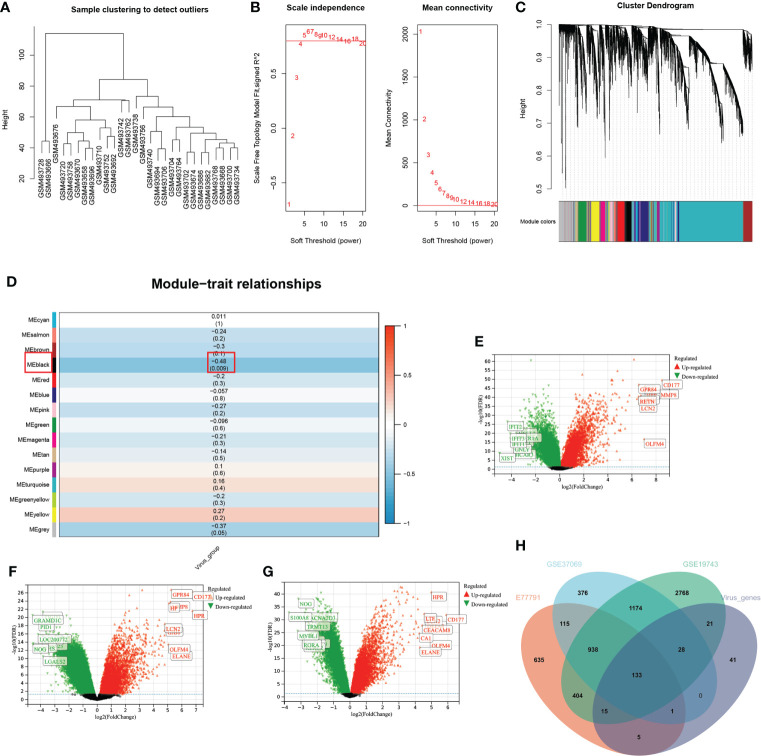
WGCNA and differential expression analysis **(A)** In the cluster analysis results of the GSE19743 dataset, abnormal samples can be eliminated according to the height value. **(B)** Analysis of scale-free fit indices (left) and average connectivity (right) to select various soft power (β). Soft threshold: select the soft threshold when R2>0.85. **(C)** Cluster dendrogram of burn trait genes; each color below represents a co-expressed gene module. **(D)** Correlation of consensus clustering groupings with gene modules. The values are the correlation coefficients (p), respectively. Red represents positive correlation, and blue represents negative correlation. Black modules have a significant correlation with virus grouping. **(E–G)**. Differential expression analysis of genes in three burn datasets. Green represents down-regulated genes, and red represents up-regulated genes. The genes in black are not significantly different. **(H)** The intersection of the three burn datasets and the black module in WGCNA.

In the WGCNA analysis, two outlier samples were excluded with a soft threshold of 16 (**Figure 4A**). The genes of GSE19743 can be divided into 14 modules (**Figure 4**). The black modules containing 244 genes were significantly correlated with VRGMPGs (*P* < 0.01, coefficient=-0.48) (**Figure 4**). In GSE19743, there were 5481 differentially expressed genes (DEGs) (|LogFC > 1|, FDR < 0.05) between burn patients and healthy adults. In GSE77791, there are 2246 DEGs. In GSE37069, there are 2765DEGs (**Figure 4G**). Finally, there were 133 intersections between the black modules of WGCNA and differently expressed genes (**Figure 4**). These genes were used in the next step of network analysis to screen the hub genes further.

### Network analysis of VRDEGs

**Figure 5:**
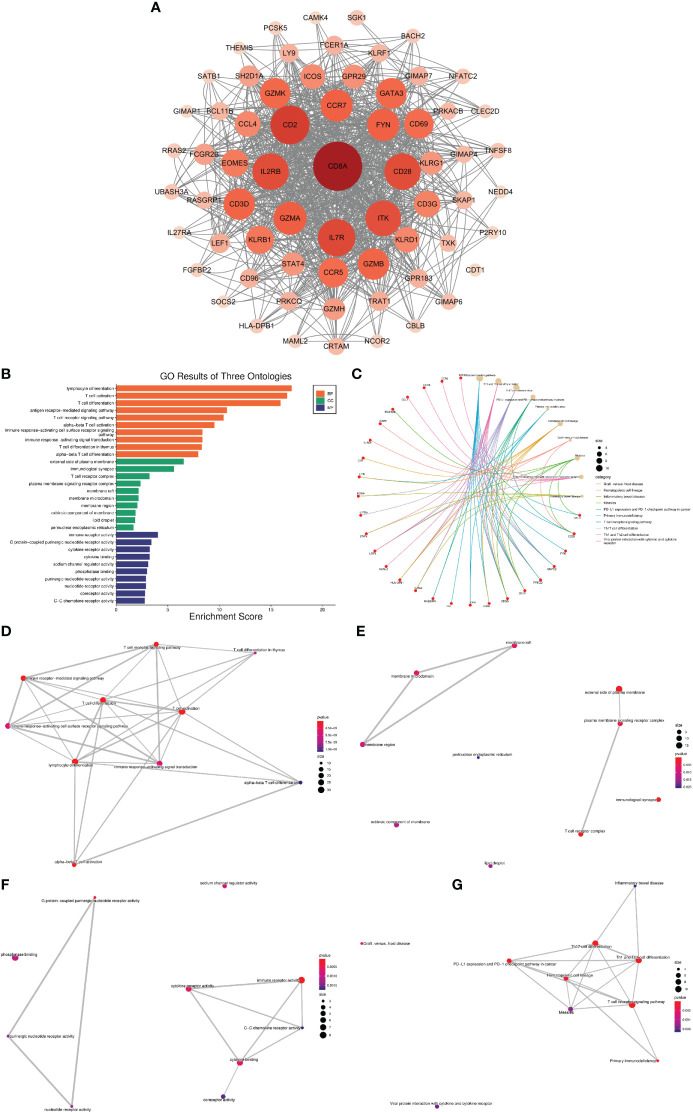
Network analysis of VRDEGs **(A)** The core gene network after the intersection of genes was screened by the MCODE plugin. The darker the color and the larger the circle, the more nodes the gene plays. **(B)** GO enrichment analysis results, orange is BP, green is CC, blue is MF, the abscissa is the enrichment score, and the ordinate is the pathway name. **(C)** KEGG enrichment analysis results. The red is the gene, and the yellow is the pathway. The larger the circle is, the more genes are enriched in the pathway. Different colors represent different enrichment results. **(D–G)**. Network plot of KEGG and GO enrichment analysis. Each link represents a commonly enriched gene between pathways, and the thicker the connecting line, the greater the number of common genes. The more enriched genes, the bigger the dots, and the smaller the P value, the redder the dots.

Using the MCODE plugin for Cytoscape, we identified 65 hub genes with dense interaction networks (**Figure 5**). In GO and KEGG enrichment analysis, these genes are highly correlated with T cell-related pathways, such as T cell activation and differentiation, T cell receptor signaling, T cell receptor complex, and Th1, Th2, and Th17 cell differentiation (**Figure 5B**). Furthermore, there are highly shared genes among these pathways, illustrating the high possibility of interaction between these genes (**Figure 5G**).

### Screening for prognosis-related genes

**Figure 6:**
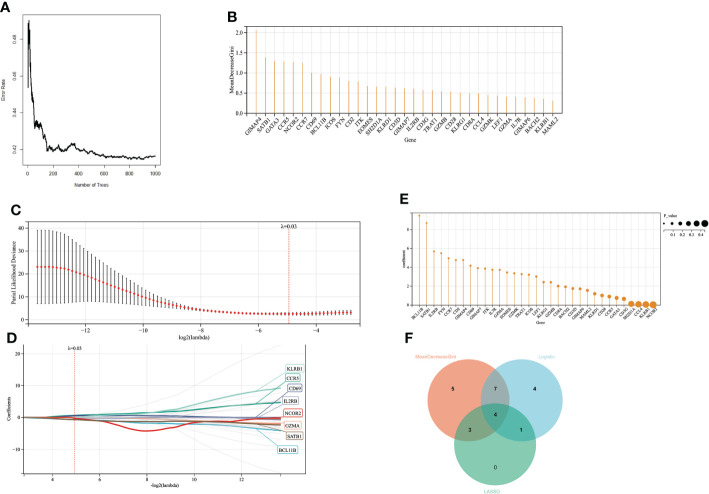
Screening variables by machine learning **(A)** When the random forest selects different numbers of decision trees, the error rate of the classification results. When the decision tree is 500, it has a lower error rate. **(B)** The ordinate is the Gini value, representing the variable’s importance in the random forest analysis. **(C, D)**. LASSO regression results have a better screening effect when the coefficient is set at 0.03. **(E)** The results of univariate logistic analysis. The ordinate is the regression coefficient, and the size of the circle is proportional to the P value. **(F)** Intersection of random forest, lasso regression, and univariate logistic regression results.

When the number of decision trees is 500, there is a lower error in RF, and the top 20 important genes are screened out (**Figure 6A**). 16 variables had non-zero coefficients in the least absolute shrinkage and selection operator (LASSO) regression model (**Figure 6C**). In GSE77791, 25 genes were significantly associated with survival (*P* < 0.05) according to the univariate analysis (**Figure 6**). Finally, we obtained 4 prognostic genes for further study (**Figure 6**).

### Constructing risk scoring models and independence verification

**Figure 7:**
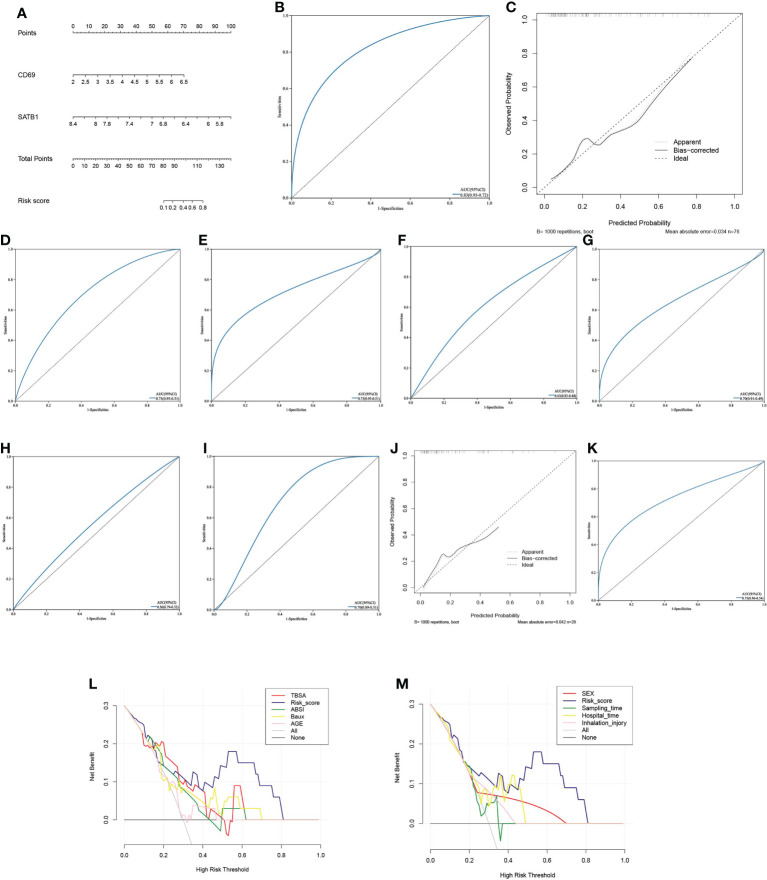
Predictive model **(A)** The Nomogram chart is constructed by multivariate logistic regression, which scores patients according to the gene value, and then predicts the risk of death. **(B)** The ROC curve of the nomogram in the training set: the larger the AUC value, the better the prediction performance. **(C)** The calibration curve of the nomogram in the training set: the higher the degree of coincidence with the diagonal line, the better the prediction performance. **(D–I)**. ROC curves of TBSA, hospital time, inhalation injury, Baux, age, and ABSI in the validation set **(J)** The calibration curve of the nomogram in the validation set. **(K)** ROC curve of the nomogram in the validation set. **(L, M)**. In the decision curve of the validation set’s nomogram, the risk cutoff value is the horizontal axis, and the larger the vertical axis, the better the prediction performance. Nomoram’s risk score has the best predictive power.

Multivariate logistic regression analysis revealed that *CD69* and *SATB1* were independent risk factors for severe burns (**Table 1**). These two independent factors were used to construct the nomogram (**Figure 7**). The AUC value of the nomogram was 0.825 (95% CI): (**Figure 7**), which indicated that the model had good predictability. Furthermore, the calibration curve showed a high consistency between prediction and actual observation (**Figure 7**). The AUCs of TBSA, hospital time, halation injury, Baux, AGE, and ABSI were 0.73, 0.73, 0.63, 0.7, 0.56, and 0.7, respectively (**Figure 7I**). The AUC of the nomogram was 0.75 in the validation set (**Figure 7**). The calibration curve also showed a relatively low consistency between prediction and actual observation (**Figure 7**). The decision curve analysis (DCA) showed that the Risk_score had the best ability to identify survival than any other clinical factor in the validation sets (**Figure 7M**). Multivariate logistic regression analysis revealed that risk scores were independent risk factors for severe burns (**Table 2**).

**Table 1:** Univariate and multivariate logistic regression for 4 genes.

	Univariate analysis			Multivariate analysis		
Genes	P	OR	95% CI	P	OR	95% CI
SATB1	0.021	0.047	(0.003-0.634)	0.008	0.025	(0.003-0.191)
CD69	0.003	10.716	(2.258-50.85)	0.001	5.001	(1.508-16.577)
BCL11B	0.008	1.456	(0.102-0.709)	0.774	–	–
IL2RB	0.026	0.111	(0.016-0.768)	0.69	–	–

**Table 2:** Univariate and multivariate logistic regression for risk score and clinical features.

Variables		Univariate analysis		Multivariate analysis		
	P	OR	95% CI	P	OR	95% CI
Risk_score	0.049	2.324	(1.003-5.383)	0.024	6.286	(0.88-44.9)
INJURY_INHALATION	0.887	1.154	(0.161-8.274)	–	–	–
TBSA	0.024	1.054	(0.986-1.127)	–	–	–
HOSPITAL TIME	0.696	1.003	(0.989-1.017)	–	–	–
SEX	0.999	–	–	–	–	–
AGE	0.622	0.947	(0.761-1.177)	–	–	–
HOURS POST INJURY	0.212	0.987	(0.967-1.008)	–	–	–
Baux	0.503	1.069	(0.88-1.299)	–	–	–
ABSI	0.823	1.258	(0.169-9.357)	–	–	–

### Immune analysis between high- and low-risk groups

**Figure 8:**
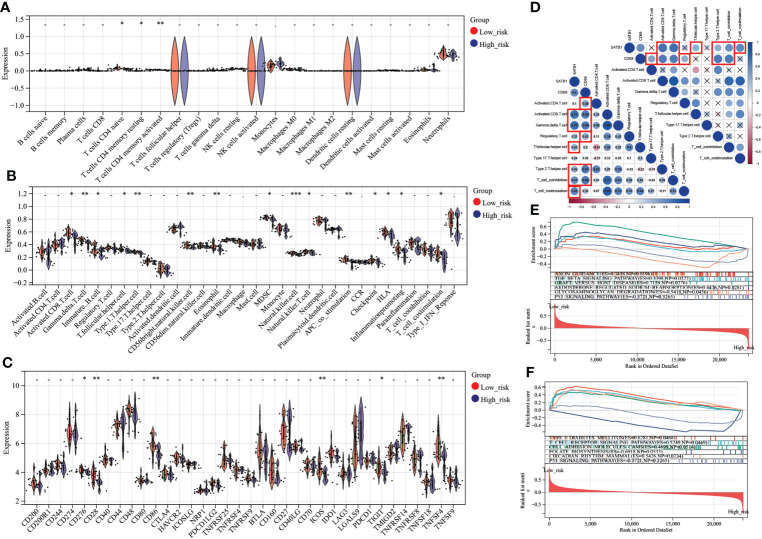
Immune analysis grouped by different risk scores **(A)** Comparison of 22 immune cells between different risk score groups. The vertical axis represents the proportion of immune cells. Cells with *P* < 0.05 were considered to be different between groups. **(B)** Comparison of immune cell and immune function scores between different risk score groups. The vertical axis is the rating. Cells with *P* < 0.05 were considered to be different between groups. **(C)** Comparison of immune checkpoint-related gene expression between different risk score groups. The vertical axis represents the gene expression level. Genes with *P* < 0.05 were considered to be different between groups. **(D)** Key genes, correlation analysis with T cell number and function. In the bottom left plot, blue represents a positive correlation, and red is a negative correlation. *P* > 0.05 is drawn as “×” in the upper right corner. Correlation coefficients > 0.4 and *P* < 0.05 are highlighted with red lines. *P* < 0.05:*, *P* < 0.01:**, *P* < 0.001:***. **(E, F)**. In the results of GSEA, different colors represent enriched pathways, and ES > 0 indicates that the genes of the low-risk group are enriched in this pathway, and ES < 0 means that the genes of the high-risk group are enriched in this pathway.

In CIBERSORT analysis, T cells *CD4* naive, T cells *CD4* memory resting, and *T* cells *CD4* memory activated were higher in the low-risk group (**Figure 8**). Similarly, in ssGSEA, the score of *CD4* T cells was higher in the low-risk group (**Figure 8**). In addition, the scores of immature B cell, *CD56* bright natural killer cell, *MDSC*, and T cell co-stimulation were also higher in the low-risk group (**Figure 8**). Interestingly, the low-risk group’s expression of immune checkpoint-related genes, such as *CD28*, *CD86*, *CD276*, *ICOS*, *TIGIT*, and *TNFSF4*, was upregulated (**Figure 8**). *CD69* significantly correlates with Activated *CD4* T cell, Activated *CD8* T cell, Gamma-delta T cell, Treg, Th2, and T cell co-inhibition. *SATB1* significantly correlates with Activated CD8 T cell, Gamma-delta T cell, T follicular helper cell, Th2, T cell co-inhibition, and T cell co-stimulation (**Figure 8**). In GSEA, Low-risk group genes were mainly enriched in AXON_GUIDANCE, TGF_BETA_ SIGNALING_PATHWAY, GRAFT_VERSUS_HOST_DISEASE, ALDOSTERONE_ REGULATED_SODIUM_REABSORPTION,TYPE_I_DIABETES_MELLITUS,T_CELL_RECEPTOR_SIGNALING_PATHWAY,CELL_ADHESION_MOLECULES_CAMS, and CIRCADIAN_RHYTHM_MAMMAL while high-risk group in GLYCOSAMINOGLYCAN_DEGRADATION,FOLATE_BIOSYNTHESIS (**Figures 8E, F**).

### Drug prediction and molecular docking

**Figure 9:**
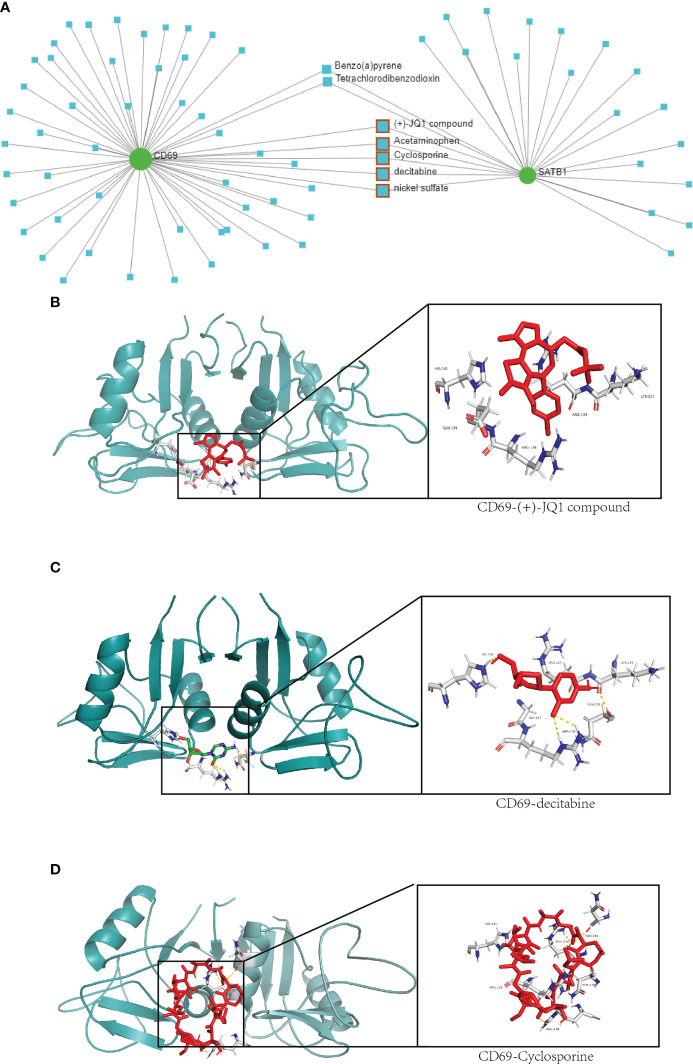
Drug network and molecular docking. **(A)** Compounds that may act on *CD69* and *SATB1* genes are predicted in the network analysis database. The compounds with therapeutic effects are selected by the red squares. **(B–D)** Molecular docking of *CD69* and JQ1, decitabine and cyclosporine compounds. The protein structure of *CD69* is in green, and the structure of the compound and its hydrogen-bonding site with *CD69* is on the right.

A total of seven chemicals were found to be effective against both *CD69* and *SATB1* (**Figure 9**). We excluded two toxic chemicals and molecularly docked five chemicals, including Acetaminophen, decitabine, Cyclosporine, NickelSulfate, and JQ1, to confirm their potential as immunosuppressive drugs for the treatment of burns. Generally, binding energy less than 0 indicates that the ligand can bind the receptor spontaneously (29); binding energy less than -5.00 kcal/mol indicates strong binding activity (30). As illustrated in **Figure 9B**, *CD69* and *SATB1* could form ligands primarily through hydrogen bonding or hydrophobic interaction. Cyclosporin, JQ1, and Decitabine performed better than the other two compounds for *CD69*. However, the binding energy of all 5 compounds was less than -5.00 kcal/mol for *SATB1*, which indicates weak binding activity (**Table 3**). The docking results could help validate the regulatory relationship between the target and the ligand.

**Table 3:** Main parameters of molecular docking of key genes and compounds, including binding energies and hydrogen bonding and hydrophobic interaction sites.

	CD69			SATB1		
	Score (kcal/mol)	hydrogen bonding	hydrophobic interaction	Score (kcal/mol)	hydrogen bonding	hydrophobic interaction
Acetaminophen	142.1	ALA136 GLU139 ARG138	TYR191 HIS141 ARG134 ASN178	262	SER117	LEU118 VAL99 LEU100
Cyclosporin	-5.2	ARG138 ARG134 ASN130	HIS141 ARG138 ASN178	-3.8	GLU97 PHE98 VAL76 VAL99 LEU10 MET113	VAL101 ALA114
Decitabine	-5.6	HIS141 ARG138 ASN178	ARG134 GLY137 LYS133	-3.6	LEU100	LEU118 SER117 GLU97 MET73
JQ1	-6.4	–	HIS141 GLU139 ARG138 ARG134 LYS133	23.6	NONE	SER117 LEU118 GLU97 MET73 LEU100 MET73
NickelSulfate	-1	–	ARG138 GLY137 ARG134 TYR135	-1	NONE	ALA114 VAL99 CYS78 VAL79 GLU97 ALA96

### Validation expression of key genes

**Figure 10:**
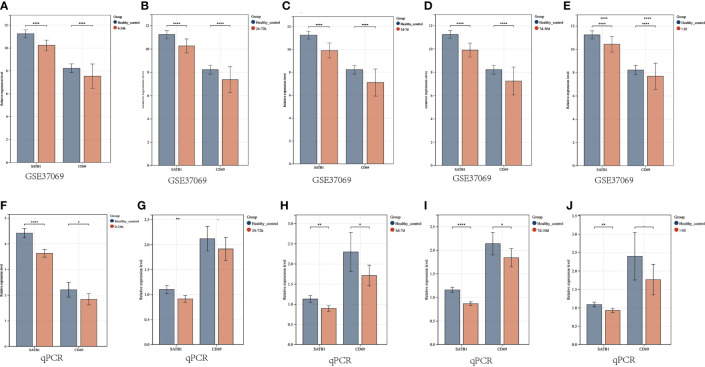
Validation of key gene expression. **(A–E)** Relative expression levels of key genes grouped by microarray at different periods (0-24h, 24-72h, 72h-7d, 7d-30d, >30d). The ordinate is the relative expression level, and the abscissa is the gene. Red bars are burn patients, and blue are normal controls. **(F–J)** Relative expression levels of key genes in PCR groups at different periods. *P* < 0.05:*, *P* < 0.01:**, *P* < 0.001:***, P>0.05: ns.

In the microarray group, *CD69* was significantly down-regulated at five time periods (0-24h, 24-72h, 72h-7d, 7d-30d, >30d), as was *SATB1*. In the PCR group, *SATB1* was significantly down-regulated at five time periods (0-24h, 24-72h, 72h-7d, 7d-30d, >30d), while there was no significant difference between burns and healthy controls at 24-72h and 30d (**Figures 10A–J**).

## Discussion

Infection and sepsis are the leading causes of death in burn patients who are often accompanied by viral infections, especially those with immunosuppression (31). However, the diagnostic, therapeutic, and prognostic value associated with the virus remains underestimated because burn patients are accompanied by fever, damaged skin structure, and immune system disturbances, which makes the virus infection less noticeable (3).

In this study, we performed a machine learning algorithm, consensus clustering, with hallmarks (related to HSV, CMV, HPV, VZV, and EBV) to divide burn patients into two virus molecular patterns (C1 vs. C2). In follow-up studies, significant differences in enrichment analysis, the ratio of immune cells, and clinical features were found between C1 and C2. It means there are different viral infection response profiles between C1 and C2 patients. In the C1 group, genes were significantly enriched in INTESTINAL_IMMUNE_NETWORK_FOR_IGA_PRODUCTION. The immune capacity of the intestinal mucosa is significantly reduced after burns. First, burns can lead to severe dysbiosis of the intestinal microbiota, reducing beneficial bacteria and increasing opportunistic pathogens (32). Secondly, the function of the intestinal mucosal barrier was damaged after burns, and bacteria invaded the blood from the intestine to induce sepsis (33). CMV latency occurs within the bone marrow, mainly within the monocyte/granulocyte progenitor cells (34), and the rate of CMV reactivation in burn patients varies from 55% to 71% (35). CMV infection reduces the immune response and exacerbates susceptibility to this bacteria (36). Our experimental results reveal the possibility of interaction between viral infection and intestinal mucosal immunity, and it will be interesting to explore further whether viral infection can exacerbate intestinal mucosal immune abnormalities.

“T_CELL_RECEPTOR_SIGNALING_PATHWAY” has been enriched in C1 groups. In addition, plasma cells, Tregs, monocytes, neutrophils, CD8 T cells, CD4 T cells, NK cells, and dendritic cells were different between VRGMPGs. After severe burns, the immune system fluctuates violently, which can generally be summarized as excessive activation of innate immune cells causing extensive inflammatory responses and immunosuppression caused by impaired adaptive immune cell function and apoptosis (37). Innate immune cells such as monocytes, neutrophils, and dendritic cells are increasing. Still, in patients with TBSA > 40%, and the ability of monocytes to migrate was damaged (38), the ability of neutrophils to phagocytosis and chemotaxis was reduced (39). The ability of dendritic cells to phagocytosis and antigen presentation was decreased (40).

Furthermore, the activation of adaptive immune response was inhibited. T cells’ landscape after burn decreased proliferation, increased apoptosis, and decreased secretion of cytokines, thereby inhibiting adaptive immunity (41, 42). In WGCNA and differential expression analysis, we obtained 133 VRDEGs significantly associated with T cell proliferation and differentiation. In GO terms, VRDEGs were mainly enriched in lymphocyte differentiation, T cell activation, and T cell activation. In KEGG analysis, VRDEGs were mainly enriched in the TCR signaling pathway, Th1, Th2, and Th17 differentiation, and viral protein interaction with cytokine and cytokine receptors. Inhibition of proliferation of T cells (especially Th cells) was a major feature of adaptive immune dysfunction after burns (43). T cells were one of the key cells against VZV infection (44), and viral infection reshapes T cell phenotype (45). Immunosuppression after burns increases the risk of VZV infection, and severe viral infection further weakens immune function and leads to death (31, 46). Inhibition of T cell also increased the risk of HSV infection. HSV induced the down-regulation of Toll-like receptor (TLR)-mediated nuclear factor-κB (NF-κB) cytokine production, which enhances further viral replication, and such patients are more susceptible to bacterial infections (4, 47). Different T cell-related pathways in two VRGMPs may be both the cause and the result of viral infection, and these mechanisms are worthy of further study. Survival and TBSA were significantly correlated with VRGMPs. The above findings illustrated that VRGMPs were associated with abnormal immune function in patients, and related therapeutic targets and prognostic markers had important prospects.

We identified the 64 hub genes related to viral infection based on the PPI network. Further, through RF, LASSO, and logistic analysis, we developed a nomogram composed of two key genes, *CD69* and *SATB1*. We confirmed that the nomogram was an independent prognostic value in multivariate logistic analysis. TBSA, ABSI, and Baux were often used to assess the severity and prognosis of burn patients (48, 49). Some researchers have also constructed prognostic models related to age, gender, length of hospital stay, and inhalation injury. Although these indicators have good prognostic value, they are often evaluated at the time of admission and cannot dynamically track the progression of burns. Changes in immune function cannot be reflected. In both training and validation sets, the AUC values of the nomogram are significantly higher (0.82 and 0.75) than those of TBSA, ABSI, and Baux, and the calibration curve shows the good performance of the nomogram. The DCA curve showed that the prognostic value of the nomogram was significantly better than any other clinical feature.

Patients could be divided into high- and low-risk groups based on the median risk score calculated from the nomogram as a cutoff. We found that CD4+T cell and CD8+T cell expression were lower in the high-risk group, which is consistent with previous findings. In addition, we also found significant differences in immune checkpoint gene expression between different risk groups. Immune checkpoint therapy was of great value in improving patient immune function and has been extensively studied in sepsis but is still unclear in severe burns. PD-L1 expression was upregulated in neutrophils and monocytes after severe burn, as was PD-1 co-inhibitory receptor expression on T cells (50, 51), which may be an important mechanism of T cell suppression. Increased IFN-γ in burn patients may be associated with increased PD-1/PDL1 expression in similar sepsis (52, 53). Anti-PD-L1 antibody therapy improves T cell suppression and survival in burnt mice (54). *PD-1* and *CTLA-4* were also a co-suppressor involved in T cell suppression in sepsis. Preclinical studies have shown that bacterial sepsis leads to increased expression of *CTLA-4* on CD4+ and CD8+ T cells, and anti-CTLA-4 treatment exhibits dose-dependent reductions in CD4+ and CD8+ T lymphocyte apoptosis and improved survival (51, 55). BTLA, another immune checkpoint inhibitor, has been associated with increased morbidity and mortality in preclinical studies (56). Increased BTLA expression on circulating CD4+ T cells in sepsis patients was associated with nosocomial infection. In a CLP mouse model of sepsis, *BTLA* knockout mice had reduced bacterial numbers, reduced organ damage markers, and improved survival (57). Immune checkpoint inhibitors have enormous value in treating burn patients with immunosuppression and sepsis. However, the current clinical efficacy is still not good, which is related to the lack of effective targets (58). Our study identified potential immune checkpoint genes in burn patients with different VRGMPs, such as *CD28*, *CD86*, *CD276*, *ICOS*, *TIGIT*, and *TNFSF4*, which were of great value for the development of new immunotherapy targets.

Immunosuppression is mainly manifested by enhanced innate immunity, such as excessive activation of neutrophils, and weakened adaptive immune responses, such as T cell apoptosis in burn patients. *CD69* and *SATB1* were significantly differentially expressed between burn patients and healthy adults. Our correlation analysis showed that its expression pattern had an important relationship with γδ T-cells, CD4/8 T cells, Th2 cells, and T cell co-inhibition.

*CD69* is a member of the C-type lectin superfamily. Once activated, *CD69* acts as a co-stimulatory molecule for T cell activation and proliferation. In burn patients, *CD69* expression was suppressed on αβ T cells, but increased on γδ T-cells in the burn wound (59). The role of *CD69* on T cell differentiation is multifaceted. Activated γδ T-cells can induce T cell subtypes to differentiate into Th2 and Th17 (60), and Th17 can inhibit the differentiation of Th1 cells, which may be an important factor in the imbalance of Th1 and Th2 differentiation after burns, and the imbalance of Th1 and Th2 differentiation is an important cause of immunosuppression. *CD69* significantly correlates with immune disorders, making it important for prognostic significance. In addition, *CD69* is an important target in regulating inflammation and immunity. Knockout of *CD69* can effectively reduce the susceptibility to inflammation caused by Th17 and play an important role for regulating immune response (61). High expression of *CD69* can promote the inhibition of T cell function while blocking *CD69* enhances the immunity of T cells. In addition to mature T cells, *CD69* is indelibly expressed by immature thymocytes, natural killer (NK) cells, monocytes, and neutrophils and is constitutively expressed by mature thymocytes. Activated NK cells also highly express *CD69*. Inhibiting NK cell function can reduce *CD69* expression and improve wound healing (62).

Similarly, high *CD69* expression was found in hyper-activated neutrophils, which mediate suppression of lymphocyte function (63). *CD69* is also associated with viral susceptibility. Activated monocytes highly express *CD69*, and activated monocytes have a higher viral load during virus infection than non-activated monocytes (64). EBV-activated specific cytotoxic T lymphocytes (CTL) highly express *CD69* and can inhibit the proliferation of lymphocytes (65). High expression of *CD69* in burn patients is associated with over-enhanced innate immunity and attenuated adaptive immune response, and this correlation gives it the ability to predict prognosis. At the same time, high *CD69* expression is associated with viral infections and is a promising therapeutic target that can improve immunosuppression in burn patients.

Special AT-rich binding protein-1 (*SATB1*) is a global chromatin organizer capable of activating or repressing gene transcription in mice and humans (66). The role of *SATB1* is pivotal for T-cell development and differentiation, with *SATB1*-knockout mice being neonatally lethal and having dysregulation of Th17 (67, 68). *SATB1*-dependent T cell activation is crucial for the correct differentiation of T cell subtypes, and inhibition of *SATB1* can inhibit Treg cell activation and differentiation (69). Apoptosis of T cells is an important factor leading to post-burn immunosuppression, and immunosuppression-induced infection leads to the death of patients (59). Our study found that *SATB1* was lowly expressed in burn patients, and the expression level of *SATB1* was significantly correlated with prognosis, demonstrating the great prognostic value of *SATB1*. *SATB1* exhibits excellent prognostic value in many diseases due to its close association with T cell development (66). However, there is still no research in the field of burns. Our study identifies the ability of *SATB1* as a prognostic marker in burn patients, and given its association with burn immunosuppression, we consider the results to be of high confidence.

Further study will be promising. Furthermore, *SATB1* is proposed to suppress transcription of *PDCD1*, encoding the immune checkpoint protein 1 (PD-1) (67). In patients with burn sepsis, *PD1* is highly expressed on immune cells, and reversing this high expression is of great help in improving immune function. In our findings, *SATB1* is down-expressed in burn patients, and reversing this underexpression is a promising immunotherapy.

Both *CD69* and *SATB1* may be involved in immunosuppression in burn patients and are promising therapeutic targets. In our study, *CD69* and *SATB1* interacted with decitabine, Cyclosporine, and JQ1. Decitabine is a chemotherapy drug used for hematological tumors. Studies have shown that decitabine can inhibit pro-inflammatory factors, which may help improve the excessive inflammatory response in burns (70). In addition, decitabine can also regulate the differentiation of T cell subtypes. Decitabine could upregulate major histocompatibility complex class I-related chains B and UL16-binding protein 1 expression, and combination treatment involving γδ T cell immunotherapy and decitabine could be used to enhance the cytotoxic killing of osteosarcoma cells by γδ T cells (71). In general, its application in burns is rare, and relevant research will be of great significance. Cyclosporine is a potent immunomodulatory agent with an increasing number of clinical applications. Its major mode of action is inhibiting the production of cytokines involved in the regulation of T-cell activation (72). Cyclosporine can inhibit *CD69*-mediated T cell activation and maturation (69), which may help regulate T cell differentiation disorders (73). However, it should be noted that systemic administration of cyclosporine can significantly suppress the immune response, which in turn induces more serious infections (31). Therefore, it is necessary to develop more precise treatment methods to explore further the therapeutic value of Cyclosporine in burn patients with immunosuppression. The Bromo- and Extra-terminal domain (BET) signaling pathway plays an important role in cell proliferation, immune responses, and pro-inflammatory events. The bromodomain inhibitor JQ1, a first-in-class potent and selective inhibitor of the Bromodomain-containing protein 4 (BRD4) signaling pathway, is widely used for various diseases (74). In sepsis, JQ1 protects the intestinal mucosal barrier and reduces levels of pro-inflammatory cytokines IL6, IL1β and IL18 (75). Over-activation of Th17 can inhibit Th1 cells (impaired in burn immunosuppression), while JQ1 impairs p300-mediated RORγt acetylation in human Th17 cells (76), which is expected to enhance the differentiation and proliferation of Th1 cells. However, JQ1 can also inhibit the function of Th1 cells from secreting IFN-γ (77). Therefore, the recovery of immune function by JQ1 is complicated, and further studies on its role in immunosuppression in burns are needed.

Viral infection in burn patients is often insidious and often misdiagnosed clinically. However, viral infection can profoundly affect the immune system of burn patients, but the crosstalk between viral infection and the immune system is currently unclear. Our study is the first to identify VRGMP in burn patients by machine learning and fully explore the differences in immune cells, immune scores, and enrichment pathways between VRGMPGs. Our study found significant differences in the activation and differentiation of T cells, especially Th cells, between VRGMPGs, which may be vital clues for diagnosis, treatment, and prognostic biomarkers. In addition, the dysfunction of Th cells is of great significance in burn patients. We believe that viral infection may affect the body’s immunity by disturbing the function of Th cells, which promotes the development of immunosuppression. Therefore, genes associated with viral molecular patterns have important prognostic and therapeutic value. We developed a reliable nomogram based on VRGs with significantly better predictive power than traditional burn indicators such as TBSA, ABSI, and Baux.

Furthermore, we predicted by network analysis and molecular docking that drugs targeting *CD69* and *SATB1* have important links to immunosuppression in burn patients. Our study also has certain limitations. First, although we identified genes associated with prognosis, the samples lacked clinical information on whether the patients were infected with the virus. If such information is available, we can construct a transcriptome-based virus diagnostic nomogram, which is important for discovering occult viral infections. Second, our study found a correlation between T cells, especially Th cells, and viral infection, but more cell and animal experiments are needed to explore the exact mechanism, which is useful for studying the mechanism between viral infection and burn immunosuppression significantly. Finally, we fully evaluated the possibility of immune checkpoint target genes and key genes as targets, which will greatly help the treatment of burn immunosuppression if they can be verified in further experiments. Overall, this study provides an overlooked perspective on post-burn viral infection and fully discusses its potential to interact with the immune system. We identified nomograms with strong prognostic, and predictive power and developed related drug targets, which have important guiding significance for future research on burn virus infection.

## Conclusion

We identified two VRGMPs in burn patients with significantly different T-cell proliferation-differentiation-related gene expression patterns and T-cell ratios. We constructed a nomogram including *CD69* and *SATB1* with stronger prognostic efficacy than common clinical indicators such as ABSI, TBSA, and Baux. In addition, we identified possible immune checkpoint inhibitor targets and immunotherapy drugs, Cyclosporin, JQ1, and Decitabine.

## Data availability statement

The datasets presented in this study can be found in online repositories. The names of the repository/repositories and accession number(s) can be found below: https://www.ncbi.nlm.nih.gov/geo/, GSE19743, GSE77791, GSE37069, GSE26440.

## Ethics statement

Ethical approval was obtained from the Ethics Committee of Xi’an Ninth Hospital (202268).

## Author contributions

PW, ZZ and RL: Consulted the literature and prepared materials. PW, ZZ, RL, JML, XZ, LJ, YW and XD: Experimented and analysed the data. PW and ZZ: Drawn up the manuscript. HX: Conceived and designed the study. HX: Financial support and final approval of the manuscript. All authors contributed to the article and approved the submitted version.

## Funding

Key Research and Development Plan of Shaanxi Province, Grant/Award Number: S2021-YF-YBSF-0936. Open Project of Provincial Key Laboratory of Union Hospital Affiliated to Fujian Medical University in 2020, Grant/Award Number: XHZDSYS202004. Xi’an Health Commission Fund Project, Grant/Award Number: 2020yb21; 2022yb03; 2022yb04; 2022yb05. China Red Cross Foundation Xu Rongxiang Regenerative Life Public Welfare Fund Research Project, Grant/Award Number: RXRL2021-05.

## Acknowledgments

We would like to thank the staff of the Department of Burns and Plastic and Cosmetic Surgery, the Ninth Affiliated Hospital of Xi’an Jiaotong University for their help in this study.

## Conflict of interest

The authors declare that the research was conducted in the absence of any commercial or financial relationships that could be construed as a potential conflict of interest.

## Publisher’s note

All claims expressed in this article are solely those of the authors and do not necessarily represent those of their affiliated organizations, or those of the publisher, the editors and the reviewers. Any product that may be evaluated in this article, or claim that may be made by its manufacturer, is not guaranteed or endorsed by the publisher.

## Abbreviations

VRGs, Virus-related genes; VRGMPs, VRGs molecular patterns; TBSA, total body surface area; VRGMPG, VRGMP groups; PAC, Proportion of ambiguous clustering; CDF, Cumulative distribution function; PCA, Principal component analysis; GSEA, Gene Set Enrichment Analysis; KEGG, Kyoto Encyclopedia of Genes and Genomes; ssGSEA, Single sample Gene Set Enrichment Analysis; PPI, Protein-protein interaction; GO, Gene Oncology; WGCNA, Weighted gene co-expression network analysis; RF, Random forest; LASSO, least absolute shrinkage and selection operator; HSV, Herpes simplex virus; VZV, varicella-zoster virus; CMV, Cytomegalovirus; HVP, human papilloma virus; EBV, Epstein-Barr virus.
